# Human immature testicular tissue organ culture: a step towards fertility preservation and restoration

**DOI:** 10.3389/fendo.2023.1242263

**Published:** 2023-08-28

**Authors:** Nagham Younis, Andre L. Caldeira-Brant, Tianjiao Chu, Shtaywy Abdalla, Kyle E. Orwig

**Affiliations:** ^1^ Department of Obstetrics, Gynecology and Reproductive Sciences, Magee-Womens Research Institute, University of Pittsburgh School of Medicine, Pittsburgh, PA, United States; ^2^ Department of Biological Sciences, School of Science, University of Jordan, Amman, Jordan

**Keywords:** *in vitro* spermatogenesis, male fertility preservation, testicular tissue cryopreservation, immature testicular tissue, gonadotoxic therapy, cancer survivorship

## Abstract

**Background:**

Cryopreservation of immature testicular tissue (ITT) is currently the only option to preserve fertility of prepubertal patients. Autologous transplantation of ITT may not be safe or appropriate for all patients. Therefore, methods to mature ITT *ex vivo* are needed.

**Objectives:**

Aim to investigate the feasibility of inducing *in vitro* spermatogenesis from ITT cryopreserved for pediatric patients prior to initiation of gonadotoxic therapy.

**Materials and methods:**

Cryopreserved-thawed ITT from prepubertal and peripubertal patients were cultured for 7, 16, and 32 days in medium with no hormones or supplemented with 5 IU/L FSH, 1 IU/L hCG, or 5IU/L FSH+1 IU/L hCG. Samples were evaluated histologically to assess tissue integrity, and immunofluorescence staining was performed to identify VASA (DDX4)+ germ cells, UCHL1+ spermatogonia, SYCP3+ spermatocytes, CREM+ spermatids, SOX9+ Sertoli cells. Proliferation (KI67) and apoptosis (CASPASE3) of germ cells and Sertoli cells were also analyzed. Sertoli and Leydig cell maturation was evaluated by AR and INSL3 expression as well as expression of the blood testis barrier protein, CLAUDIN11, and testosterone secretion in the culture medium.

**Results:**

Integrity of seminiferous tubules, VASA+ germ cells and SOX9+ Sertoli cells were maintained up to 32 days. The number of VASA+ germ cells was consistently higher in the peripubertal groups. UCHL1+ undifferentiated spermatogonia and SOX9+ Sertoli cell proliferation was confirmed in most samples. SYCP3+ primary spermatocytes began to appear by day 16 in both age groups. Sertoli cell maturation was demonstrated by AR expression but the expression of CLAUDIN11 was disorganized. Presence of mature and functional Leydig cells was verified by INSL3 expression and secretion of testosterone. Gonadotropin treatments did not consistently impact the number or proliferation of germ cells or somatic cells, but FSH was necessary to increase testosterone secretion over time in prepubertal samples.

**Conclusion:**

ITT were maintained in organotypic culture for up to 32 days and spermatogonia differentiated to produce primary spermatocytes in both pre- and peripubertal age groups. However, complete spermatogenesis was not observed in either group.

## Introduction

Treatments for childhood cancers are improving with post-therapy survival rates of 88% in 2021 (1), which means most young patients can look forward to a full and productive life after cure, including the possibility of having children. However, an unfortunate side effect of cancer therapy is the loss of fertility, since treatments target not only the rapidly dividing cancer cells, but also the proliferating germ cells in the gonads of those patients (2, 3). Infertility rates among childhood cancer survivors can range from 42-66% (4). Young adult survivors of childhood cancers experience distress about the potential for infertility (5–7), and desire to have offspring from their own cells, especially those with the religious restrictions of using donated sperm in some cultures (8). Therefore, the American Society for Clinical Oncology (9), the American Society for Reproductive Medicine (10) and the International Society for Fertility Preservation (11) recommend that all patients are counseled about the reproductive side effects associated with treatment of their primary disease as well as options to preserve fertility. Adult patients have the option to cryopreserve oocytes or sperm prior to treatment that can be thawed in the future and used to achieve pregnancy with established assisted reproductive technologies (12–14). The only fertility preservation options available to prepubertal children who are not yet producing mature oocytes or sperm are ovarian tissue or testicular tissue cryobanking (15, 16).

Although immature testicular tissue (ITT) from prepubertal boys do not produce sperm, they do contain spermatogonial stem cells (SSCs) that have the potential to produce sperm using one of several methods that are in the research pipeline (15). Autologous spermatogonial stem cell transplantation and testicular tissue grafting are mature technologies that have been replicated in numerous mammalian species (reviewed in (15)), and may be ready for translation to the human clinic. However, these autologous transplantation approaches may not be safe for patients with leukemia, testicular cancer or metastasizing disease due to concerns about reintroducing malignant cells back into patient survivors. Autologous transplantation may also be undesirable to transgender patients who do not want to be exposed to elevated testosterone levels that may be necessary to mature their tissues and produce sperm inside their bodies. Therefore, methods are needed to mature ITT outside the patient’s body and produce sperm. Xenografting ITT into an animal host such as mice, pigs or primates, to mature and produce sperm is a promising option (17) but raises concerns about the potential risk of exposing patients to xenobiotic diseases (18). Furthermore, exposure of ITT to porcine or bovine hosts will not be acceptable in some religions (19, 20). Methods are needed to mature ITT outside the patient’s body.

Sato and colleagues pioneered the method of ITT organotypic culture in 2011 in mice. Immature testicular tissue was cultured at the air/liquid interface on an island of agar that was half soaked in culture medium and matured over 30-40 days to produce sperm and live offspring (21). Over the past decade, the same group has continually optimized the approach using microfluidic devices to maintain tissues viable with continuous sperm production over several months (22–24). Critical features of the culture that supported long-term spermatogenesis in mouse organotypic culture were the replacement of FBS with knockout serum replacement (KSR) or Albumax, a lipid rich subfraction of KSR (23, 25, 26). Others have replicated the Ogawa testicular tissue organ culture methods with production of spermatids or sperm (27, 28) but production of offspring has not been replicated by any other research group in mice and translation to other species, including humans, remains a work in progress (see below).

Several groups have replicated the air/liquid interface culture with human ITT and reported variable results. Medrano and co-workers tested KSR versus FSH, 37°C versus 34°C, and with or without follicle stimulating hormone (FSH) and luteinizing hormone (LH) (Menopur 75, Ferring) and reported that cultures maintained at 34°C with KSR exhibited the best survival of spermatogonia and Sertoli cells. The addition of LH and FSH improved Sertoli cell survival and promoted the maturation of germ cells up to the initiation of meiosis (SYCP3+ cells) but not beyond (29). Portela and colleagues used media supplemented with retinoic acid and melatonin, with or without FSH/LH. Spermatogonia survived and proliferated but the overall number of spermatogonia decreased over five weeks in culture and spermatogenesis was not initiated. Sertoli cells exhibited transient proliferation but did not mature to express the androgen receptor (AR). Culture outcomes were not impacted by cryopreservation or the addition of LH/FSH (30). Wang and colleagues used media supplemented with an extensive cocktail of growth factors, with or without retinoic acid (RA) (31). BOL+ spermatocytes were observed in the condition with RA on day 60 of culture, but those results were not quantified.

De Michele and colleagues used a variation of testis organotypic culture in which ITT was maintained in transwells at 34°C in an enriched DMEM-F12 medium supplemented with 10% KSR and 5 IU/L FSH. Viability and integrity of seminiferous tubules was maintained for 139 days, including the production of meiotic and post-meiotic cells. Spermatogonial proliferation and numbers decreased over time in culture. Sertoli cell numbers remained constant, but proliferation decreased over time in culture, which is typical of mature Sertoli cells, but AR expression did not change during culture. Leydig cell maturation was demonstrated by increased STAR expression and secretion of testosterone, which declined over time in culture. Supplementation of the culture medium with a cocktail of human chorionic gonadotropin (hCG), glial cell derived neurotrophic factor (GDNF), vitamin A and C, hydroxycholesterol, and triiodothyronine (T3) did not improve outcomes (32).

In summary, methods and results of organotypic culture with human immature testicular tissues are inconsistent and robust development of a complete seminiferous epithelium has not yet been achieved. Maturation of the testicular niche is likely critical to supporting complete spermatogenesis. LH and FSH are important for the maturation of Leydig cells and LH stimulates testosterone production, which is required for spermatogenesis. FSH supports the maturation of Sertoli cells that nurture every stage of spermatogenic lineage development and mediate the effects of testosterone (33–36). The studies described above included FSH and/or LH (or human chorionic gonadotropin, hCG) in some or all of their cultures but did not individually test the importance of these gonadotropins. The De Michele study reported the most advanced germ cell development with sporadic progression through meiosis and production of haploid cells. Therefore, we will attempt to replicate the De Michele approach (32, 37) and test the importance of FSH and LH, individually and in combination. Finally, we tested the hypothesis that Sertoli cells and Leydig cells in tissues from peripubertal patients are already mature and may be poised to support development of a spermatogenic epithelium.

## Materials and methods

### Human tissues

**Figure 1:**
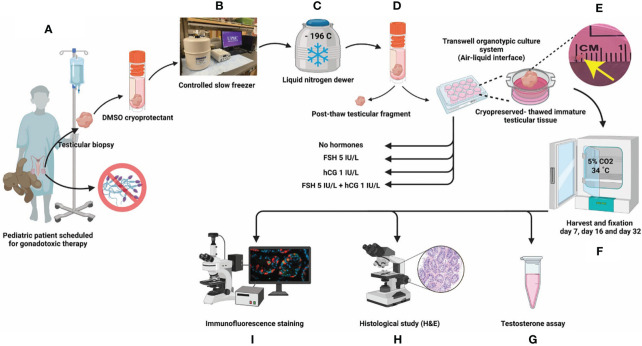
Experimental scheme. **(A)** ITT was collected by biopsy from patients before the initiation of gonadotoxic therapy and **(B)** cryopreserved by computer controlled slow rate freezing for **(C)** long term cryostorage in liquid nitrogen. Each patient donates 25% of their tissue to research. **(D)**. For this study, ITTs were thawed from three prepubertal patients (1-3 y/o) and three peripubertal patients (9-12 y/o). **(E)** ITT was cultured in DMEM/F12 media supplemented with 10% KSR and one of four *in vitro* treatments (none, FSH, hCG and FSH + hCG). **(F)** Cultured ITT was harvested on days 7, 16 and 32 of culture. **(G)** Culture media was assayed for testosterone. **(H)** Tissues were fixed for histology (hematoxylin and eosin) and **(I)** immunofluorescence.

The UPMC Fertility Preservation Program (https://fertilitypreservationpittsburgh.org/) has cryopreserved >700 ITT since 2011 for patients who were at risk of infertility due to their diseases or medical treatments. Briefly, testicular tissues were obtained by a wedge biopsy comprising about 20% of one testis. Testicular parenchyma was dissected from any adhering tunica and cut into small pieces measuring 2-5 mm in diameter. Tissues are allocated 75% for the patient’s future reproductive use and 25% to research; and cryopreserved in modified human tubal fluid containing 5% DMSO and 5% serum substitute supplement (SSS) (38). Research tissues were deidentified and transported to the research laboratory for cryostorage and experimentation (**Figures 1A–C**). All human subjects research was reviewed and approved by the University of Pittsburgh Institutional review board (IRB, STUDY19020220 and STUDY19110083) and registered with clinicaltrials.gov (NCT02972801).

### Study design

For the current study ITT from six patients was thawed as previously described (39) and allocated to prepubertal (ages 1, 2 and 3) and peripubertal (ages 9, 11 and 12) groups (**Table 1**). We excluded any patients who had previous gonadotoxic therapy, patients with potential malignant cells in their ITT (e.g., testicular cancer, leukemia) and patients with differences in sexual development (**Table 1**). Selected cryopreserved ITT samples were removed from liquid nitrogen dewars and thawed rapidly in a 37°C water bath, as described previously (39) (**Figures 1C, D**). Thawed testicular tissue for each patient was divided into five small fragments (~1 mm³) when possible (see explanation for missing data in **Supplementary Table 1**). One ITT fragment was fixed in 4% paraformaldehyde (PFA) overnight and labeled as post-thaw sample and four fragments were placed in organotypic culture at the air-liquid interface as previously described by De Michele and colleagues (32). Briefly, ITTs were placed in 12 mm diameter/0.4 µm polycarbonate membrane Transwell^®^ inserts (Corning^®^ Incorporated) and cultured in a 5% CO2 humidified incubator at 34°C for 7, 16 or 32 days. The culture medium, CTS™ KnockOut™ DMEM/F-12 culture medium (Cat. No. A1370801, Thermofisher Scientific) supplemented with 10% CTS Knockout SR xenofree medium (KSR, Cat. No. 12618012, Thermofisher Scientific) and 1% penicillin-streptomycin (Cat. No. 15140122, Thermofisher, Gibco), was refreshed every 48 hours. ITT fragments of each patient were cultured under four conditions: 1) no gonadotropins, 2) 5 IU/L FSH (Gonal-F 75 IU, Merck Serono), 3) 1 IU/L hCG (Cat. No. CG5-1VL, lyophilized powder, vial of ~5,000 IU, Sigma-Aldrich) or 4) 5 IU/L FSH + 1 IU/L hCG, starting from day 2 (**Figures 1D, E**). Tissues from an individual patient were divided among the four gonadotropin treatment groups (no gonadotropins, FSH, hCG, FSH + hCG). There was not enough tissue from individual patients to also spread across the three culture timepoints. Therefore, one prepubertal sample (1 y/o) and one peripubertal sample (9 y/o) were analyzed on culture day 7; a second prepubertal (2 y/o) and second peripubertal (11 y/o) sample were analyzed on culture day 16 and a third prepubertal (3 y/o) and third peripubertal (12 y/o) sample were analyzed on culture day 32. Tissues from both pre- and peripubertal groups and each gonadotropin treatment group were collected on days 7, 16 and 32 of culture; fixed in 4% PFA overnight and paraffin embedded for histology and immunofluorescence staining (**Figures 1F, H, I**). Spent media was collected on days 4, 8 and 12 of culture and assayed for testosterone (**Figure 1G**). The testosterone experiment required additional samples because the d8 and d12 timepoints could not be collected from the cultures that were terminated on day 7 (**Supplementary Table 2**).

**Table 1:** Patient groups for histology and IHC studies.

Pubertal category	Patient	Age (years)	Diagnosis	Previous treatment
Prepubertal	P1	1	Mucopolysaccharidosis (MPS)1	None
	P2	2	Chronic granulomatous disease	None
	P3	3	X linked chronic granulomatous disease	None
Peripubertal	P4	9	Rhabdomyosarcoma (prostate)	None
	P5	11	Chronic granulomatous disease	None
	P6	12	Ewing’s sarcoma of fibula	None

### Histological analysis and immunofluorescence staining

**Figure 2:**
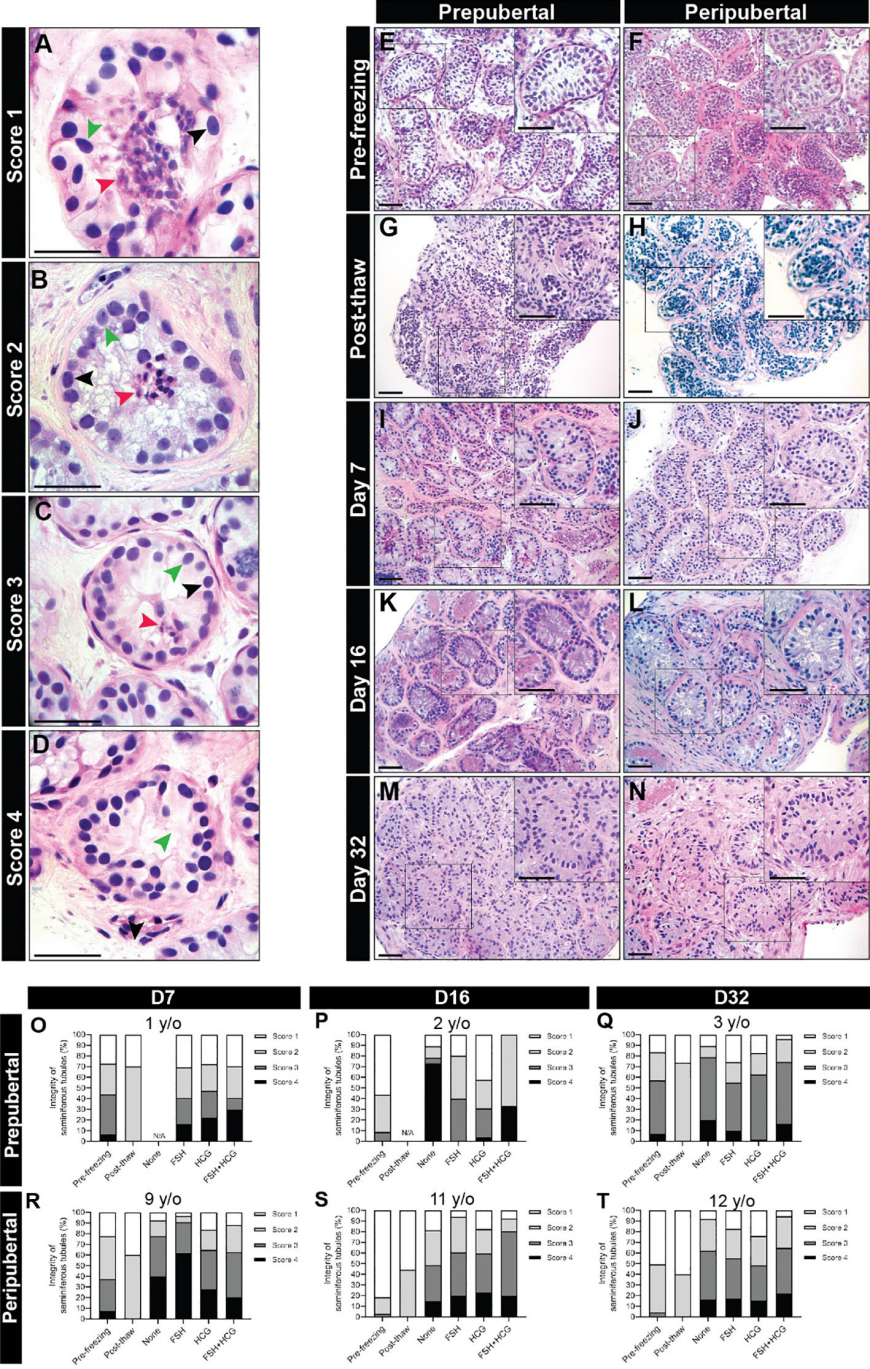
Scoring seminiferous tubule histological integrity. **(A–D)** Scoring system was used to classify the testicular samples as having poorly preserved **(A)**, score 1), fair **(B)**, score 2), good **(C)**, score 3) or best preserved **(D)**, score 4) histology. Black, green, and red arrowheads indicate spermatogonia, Sertoli cell, and pycnotic/apoptotic nuclei, respectively. Scale bars: 50 µm. **(E–N)** Hematoxylin and eosin-stained photomicrographs of immature testicular tissue from prepubertal and peripubertal patients at different time points: pre-freezing **(E, F)**, post-thaw **(G, H)**, and days 7 **(I, J)**, 16 **(K, L)** and 32 **(M, N)** *in vitro*. Scale bars: 100 µm. **(O–T)** Seminiferous tubules integrity classification based on scoring system from prepubertal **(O–Q)** and peripubertal patients **(R–T)** of pre- freezing, post-thaw, and four *in vitro* conditions (none, FSH, hCG and FSH + hCG), at different time points (day 7, 16 and 32).

Cultured testicular samples were harvested at each designated time point and fixed in 4% PFA overnight at 4C. The tissues were washed three times with D-PBS, embedded in paraffin and cut into 5 µm sections. Each tissue fragment was serially sectioned. A minimum of three non-consecutive sections (separated by 50 um or more) were analyzed for each experiment. Hematoxylin and eosin (H&E) staining was performed to assess tissue integrity under light microscopy. Integrity of seminiferous tubules was evaluated and divided into four scores as previously described (32, 39) (**Figures 2A–D**), where score 4 has the best tubular integrity and score 1 has the worst.

For immunofluorescence (IF) staining, the slides were warmed at 60°C and deparaffinized with 2X xylene, 5 minutes each. Sections were rehydrated in graded ethanol series: 2X 100% EtOH, 10 min; 95% EtOH, 5 min; 80% EtOH, 5 min; 70% EtOH, 5 min; 50% EtOH, 5 min; 25% EtOH, 5 min; PBS, 3 min. Antigen retreivel was performed in a 97°C water bath for 30 minutes using either sodium citrate (pH 6) or Tris (pH 10) buffers. Sections were blocked with 5% donkey serum for at least one hour and incubated with primary antibodies overnight. Different antibodies were used to characterize different stages and activities of germ and somatic cells. Goat anti-human DDX4 (VASA, 1:100, AF2030 Thermo Scientific) was used as a broad germ cell marker, goat or mouse anti-human UCHL1 (1:200, LS-B16043-50 or 7863-1004 Bio-Rad) for undifferentiated spermatogonia, rabbit anti-human SYCP3 (1:300, NB300-232SS Novus Biologicals) for primary spermatocytes, rabbit anti-human CREM (1:100, LS-B13702-50) for early spermatids, mouse anti-human KI67 (1:50 550609 BD Biosciences) for proliferation, rabbit anti-human cleaved CASPASE 3 (1:300, 96615 Cell Signaling Technology) for apoptosis, rabbit or goat anti-human SOX9 (1:400 Ab5535, Fisher Scientific or 1:100 AF3075 R&D) for Sertoli cells, rabbit anti-human AR (1:100 Ab271891 Abcam) for mature Sertoli cell, rabbit anti-human CLAUDIN 11 (1:100 36-4500 Invitrogen) for tight junctions of blood-testis barrier and rabbit anti-human INSL3 (1:1500, HPA028615 Sigma) for mature Leydig cells. After 24 hours of incubation with primary antibodies, the slides were rinsed with Phosphate-buffered saline-Tween 20 (PBST) twice for 5 minutes each. Then the sections were incubated with secondary antibodies using Alexa Fluor^®^ secondary antibodies conjugates 488, 568 or 647, (1:200, Invitrogen) for 45 minutes at room temperature. All seminiferous tubules with a clearly demarcated basement membrane were included in the analyses without bias. An average 60 of cross-sections or longitudinal sections of seminiferous cords/tubules were measured, totaling an area of 947,000 +/- 496,000 μm2 per patient. Negative controls were stained with isotype control antibodies. Positive controls used adult human testicular tissues that contain complete spermatogenesis (**Supplementary Figure 3**). All slides were mounted with mounting medium containing DAPI (H-2000-10, Vector labs).

### Testosterone assay

The supernatants of spent culture media were collected from 12 patients from both age groups at three time points: day 4, day 8 and day 12 and stored at -20C. Testosterone level was measured at the University of Virginia Center for Research in Reproduction Ligand Assay and Analysis Core (https://med.virginia.edu/research-in-reproduction/ligand-assay-analysis-core/), using enzyme-linked immunosorbent assay (ELISA) (IBL-America).

### Statistical analysis

For statistical analyses, all data are log2 transformed. For count data where 0 entries are present, 1 is added to all counts before the log2 transformation. All comparisons were based on linear regression models. When multiple comparisons were performed simultaneously, Tukey’s *post hoc* tests were applied to control the familywise error rates.

## Results

### ITT histological evaluation

Seminiferous tubules from prepubertal and peripubertal groups showed a disorganized pattern post-thawing, with several pyknotic cells, indicated by lower integrity scores of 1-2 (compare pre-freezing in **Figures 2E, F** to post-thaw in **Figures 2G, H**). After 7 days of culture, germ and somatic cell organization and adherence to the basement membrane improved (**Figures 2I-N**), and high scores were assigned for all ITT fragments from 7-32 days *in vitro* (**Figures 2O–T**). We did not observe any obvious differences in the histology score of prepubertal versus peripubertal group during culture. Gonadotropin treatments did not appear to impact the histology score in the prepubertal or peripubertal groups during culture (**Figures 2O–T**).

### Number of germ cells

**Figure 3:**
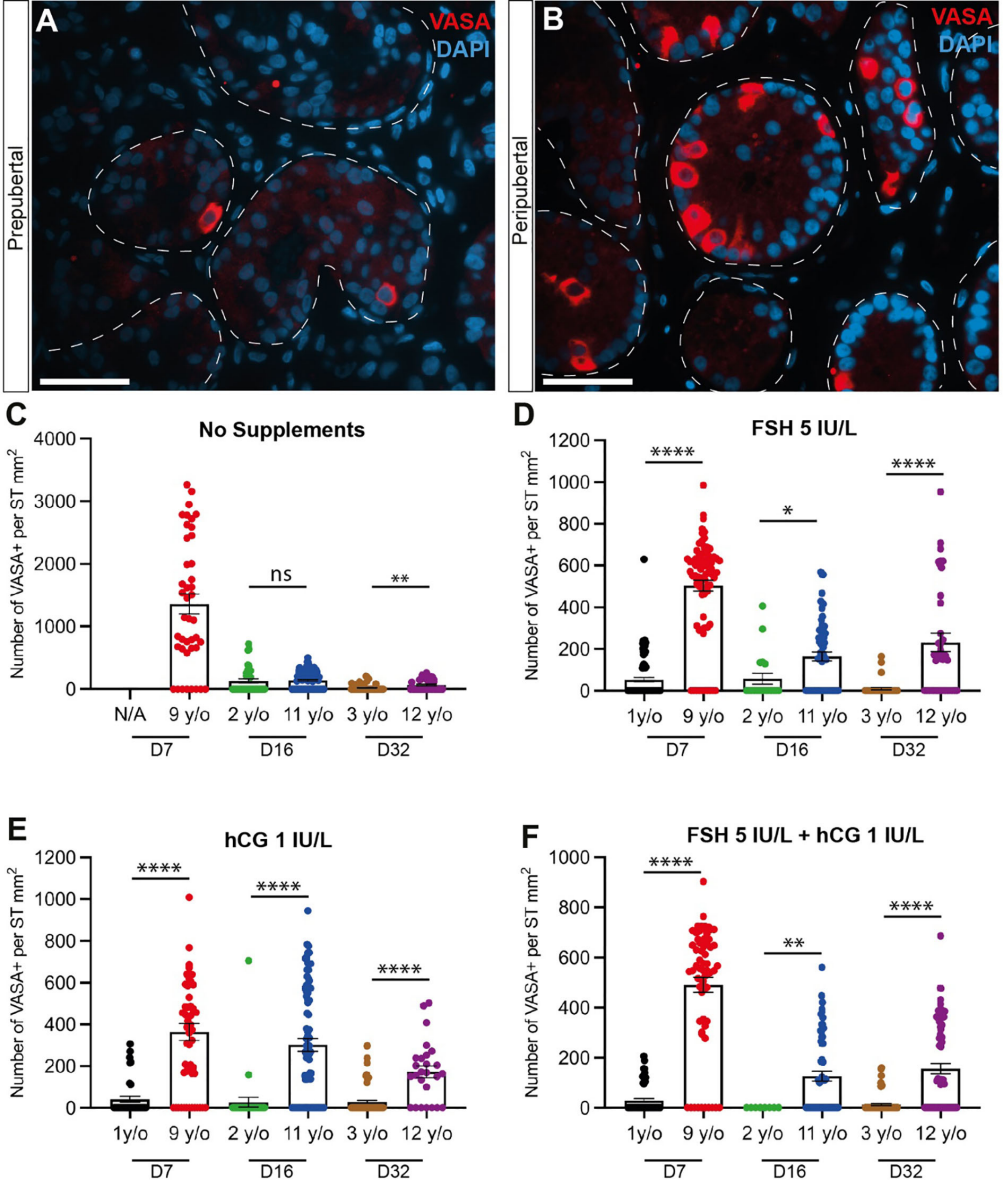
Quantification of germ cells. **(A, B)** Immunofluorescence staining for pan-germ cell marker, VASA in prepubertal **(A)** and peripubertal **(B)** age groups. Scale bars: 50 µm. Dashed white line: basal lamina. **(C–F)** Number of VASA+ germ cells per mm² of seminiferous tubule in prepubertal and peripubertal patients on day 7, 16 and 32 in four *in vitro* hormonal conditions, none **(C)**, FSH **(D)**, hCG **(E)** and FSH+ hCG **(F)**. Statistically significant differences are indicated (*p ≤ 0.05, **p ≤ 0.01, ****, p ≤ 0.0001). Raw data VASA+ germ cell numbers for post-thaw day 0 as well as days 7, 16 and 32 of culture are shown in **Supplementary Table 3**.

Immunofluorescence staining confirmed the presence of VASA+ germ cells in both prepubertal (**Figure 3A**) and peripubertal (**Figure 3B**) groups and that germ cells were maintained up to 32 days (**Figures 3C-F**). The number of germ cells in peripubertal tissue was significantly greater than in samples from prepubertal patients in almost every treatment group and at each culture timepoint (**Figures 3C-F**). Individual values for VASA+ cells in each treatment group at each culture timepoint, including post-thaw day 0 are in **Supplementary Table 3**. No consistent effect of gonadotropin treatment was observed in either group. There was a trend of decreasing germ cell numbers during the 32-day culture period, which was statistically significant in most culture conditions (**Supplementary Figure 1**).

### Undifferentiated spermatogonia number, proliferation and apoptosis

**Figure 4:**
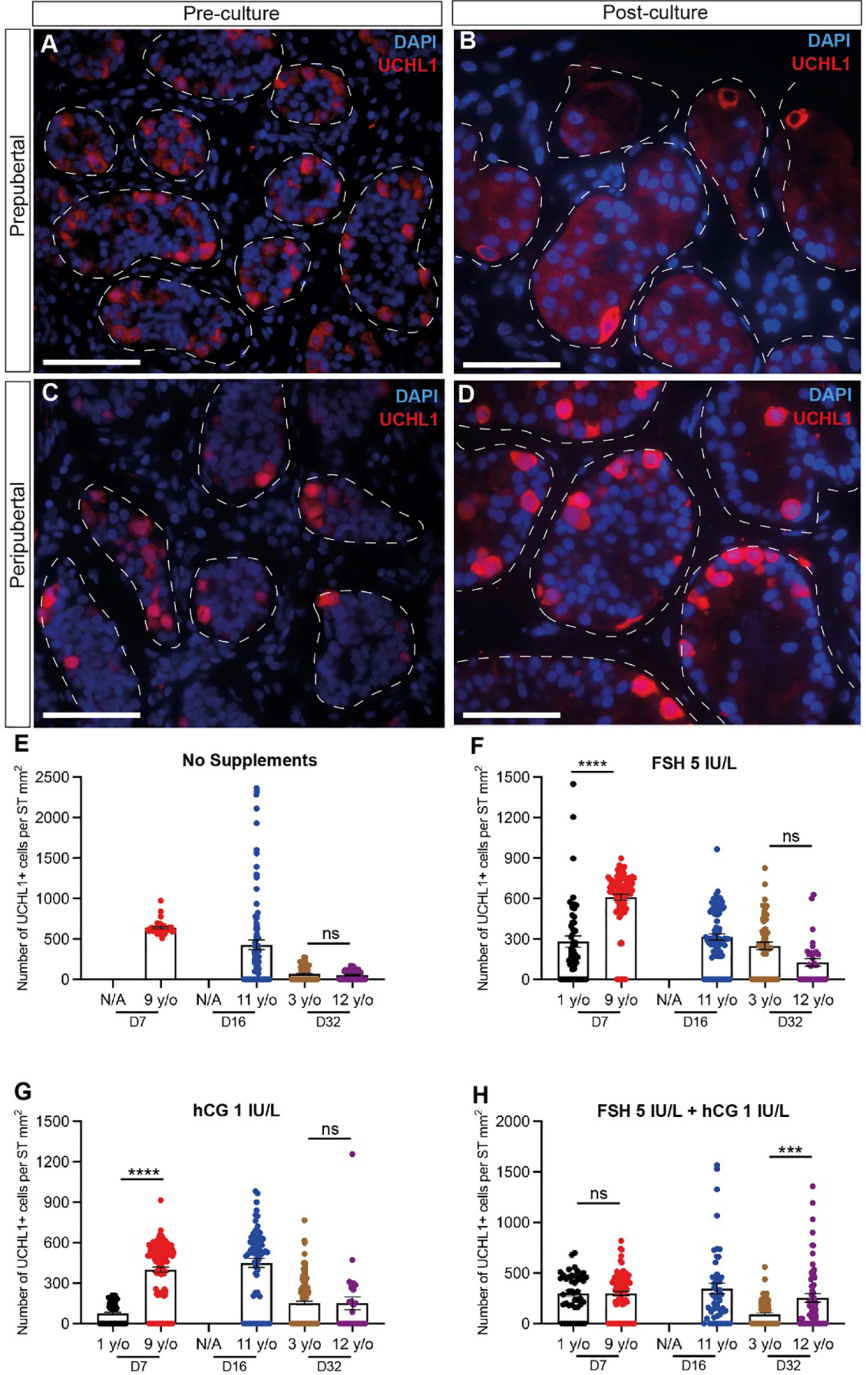
Quantification of undifferentiated spermatogonia. **(A–D)** Immunofluorescence staining for UCHL1+ undifferentiated spermatogonia in pre cultured **(A, C)** and post-cultured **(B, D)** ITTs in prepubertal **(A, B)** and peripubertal **(C, D)** groups. Dashed white line: basal lamina. Scale bars: 50 µm. **(E-H)** Number of UCHL1+ spermatogonial cells per mm² of seminiferous tubule in prepubertal and peripubertal patients on day 7, 16 and 32 of culture in four *in vitro* conditions, none **(E)**, FSH **(F)**, hCG **(G)** and FSH + hCG **(H)**. Statistically significant differences are indicated (***p ≤ 0.001, ****p ≤ 0.0001). Raw data UCHL1+ germ cell numbers for post-thaw day 0 as well as days 7, 16 and 32 of culture are shown in **Supplementary Table 3**.

**Figure 5:**
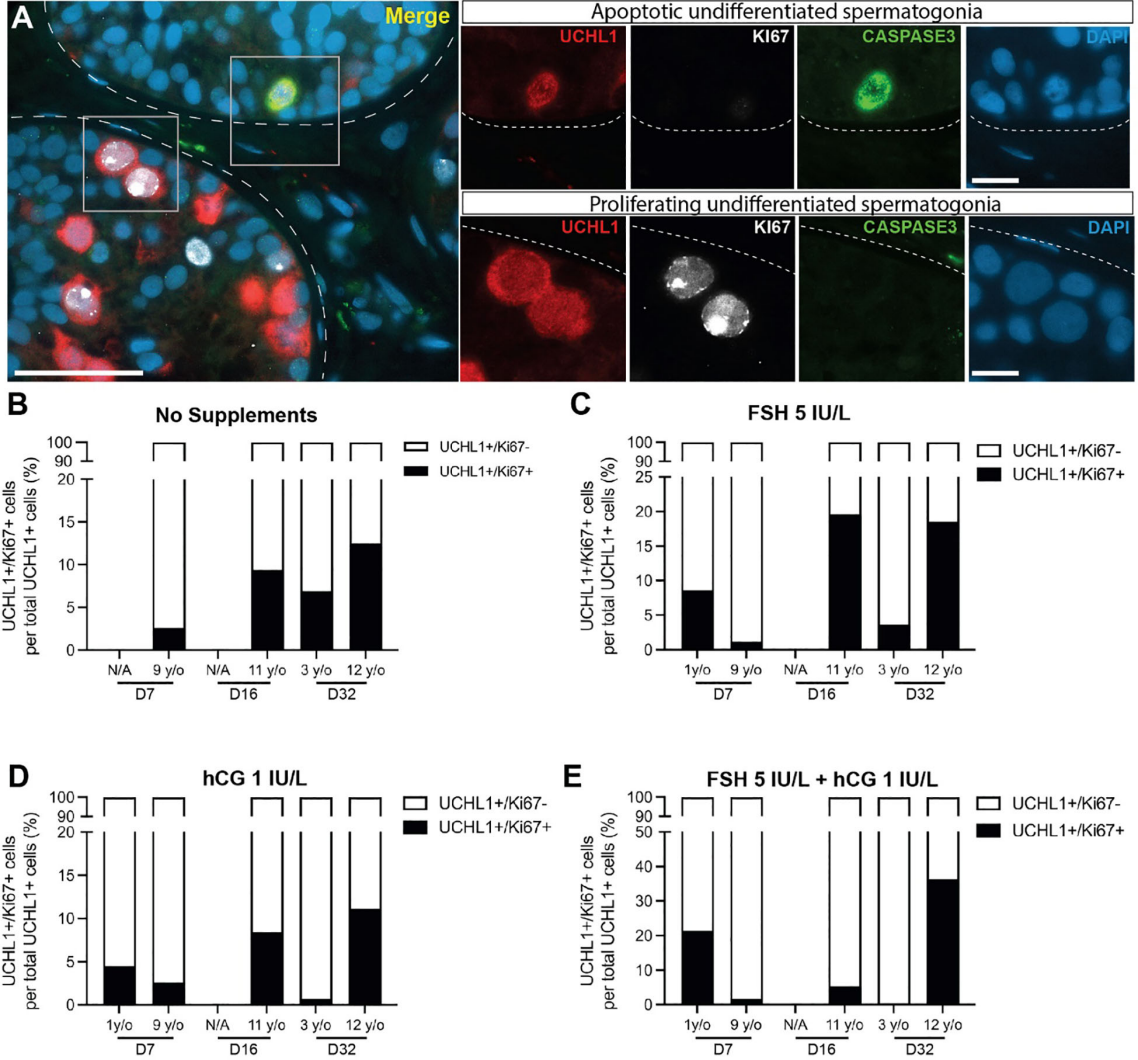
Proliferation and apoptosis of undifferentiated spermatogonia. **(A)** Immunofluorescence co-staining of UCHL1+ undifferentiated spermatogonia, proliferation marker, KI67, and apoptosis marker, CASPASE3. Dashed white line: basal lamina. Scale bars: 50 µm for A and 10 µm for high magnification single channel panel breakouts. **(B–E)** UCHL1+/KI67+ proliferating spermatogonia as a percentage of total UCHL1+ spermatogonia in prepubertal and peripubertal patients on day 7, 16 and 32 in four *in vitro* conditions, none **(B)**, FSH **(C)**, hCG **(D)** and FSH + hCG **(E)**. Raw quantitative data for the number of UCHL1+ spermatogonia in each treatment condition of the two age groups at different time points are shown in **Figure 4**.

UCHL1+ undifferentiated spermatogonia were observed in prepubertal and peripubertal tissues (**Figures 4A-D**) and their numbers were not consistently impacted by treatment or time in culture (**Figures 4E-H**). Proliferation (KI67+) and apoptosis (CASPASE 3+) of undifferentiated spermatogonia (UCHL1+) were assessed by immunofluorescent co-staining (**Figure 5A**). Proliferating spermatogonia (UCHL1+/KI67+) were observed in both age groups and all treatment conditions representing 0.5-30% of total UCHL1+ spermatogonia. Individual values for UCHL1+ cells in each treatment group at each culture timepoint, including post-thaw day 0 are in **Supplementary Table 3**. Gonadotropin treatments did not have a consistent effect on spermatogonial proliferation in either age group (p>0.05, **Figures 5B-E**). Caspase3+ apoptotic spermatogonia were rarely observed in in any culture condition or in either age group (data not shown).

### Spermatogonial differentiation

**Figure 6:**
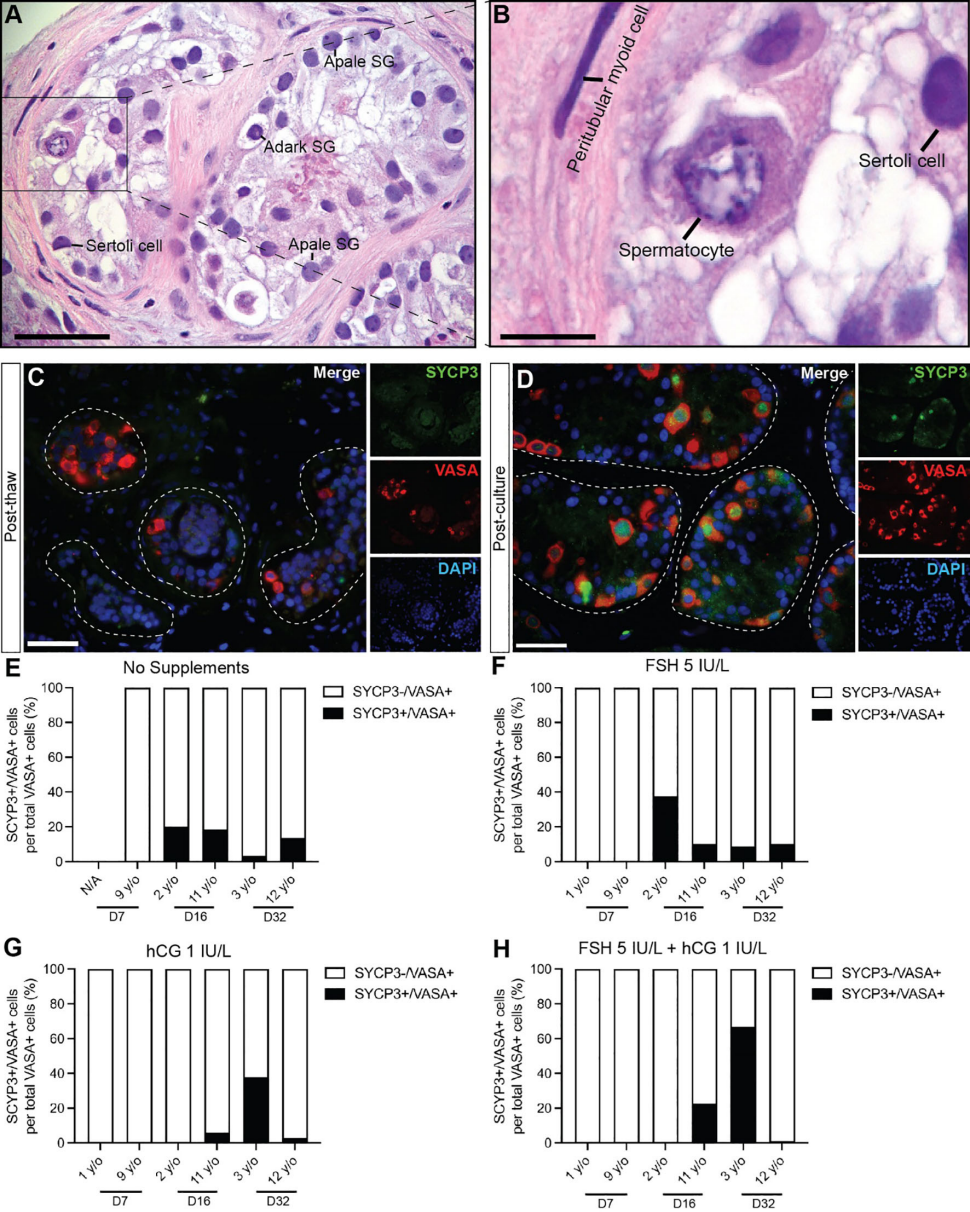
Spermatogonial differentiation. **(A, B)** Hematoxylin and eosin staining of cultured immature testicular tissue showing the presence of primary spermatocytes. Scale bars: 50 µm **(C, D)** Immunofluorescence of SYCP3+ primary spermatocytes and VASA+ germ cells of post thaw ITT **(C)** and post cultured ITT **(D)**. Dashed white line: basal lamina. Scale bars: 50 µm. **(E–H)** Percentage of SYCP3+ primary spermatocytes per total number of VASA+ germ cells in prepubertal and peripubertal patients on day 7, 16 and 32 in four *in vitro* conditions, none **(E)**, FSH **(F)**, hCG **(G)** and FSH + hCG **(H)**. Raw data VASA+/SYCP3+ germ cell numbers for post-thaw day 0 as well as days 7, 16 and 32 of culture are shown in **Supplementary Table 3**.

Spermatogonial differentiation and initiation of meiosis was indicated by the appearance of SYCP3+ cells in most samples on day 16 and day 32 in both prepubertal and peripubertal ITT cultures (**Figure 6**). No SYCP3+ cells were observed in the pre-culture (post-thaw, **Figure 6C**) or day 7 culture samples (**Figures 6E-H**) in either age group. Individual values for SYCP3+/VASA+ cells in each treatment group at each culture timepoint, including post-thaw day 0 are in **Supplementary Table 3**. Gonadotropin treatment did not impact the appearance of SYCP3+ cells. We did not observe CREM+ early spermatids in either age group or any culture condition.

### Sertoli cell number, proliferation and apoptosis

**Figure 7:**
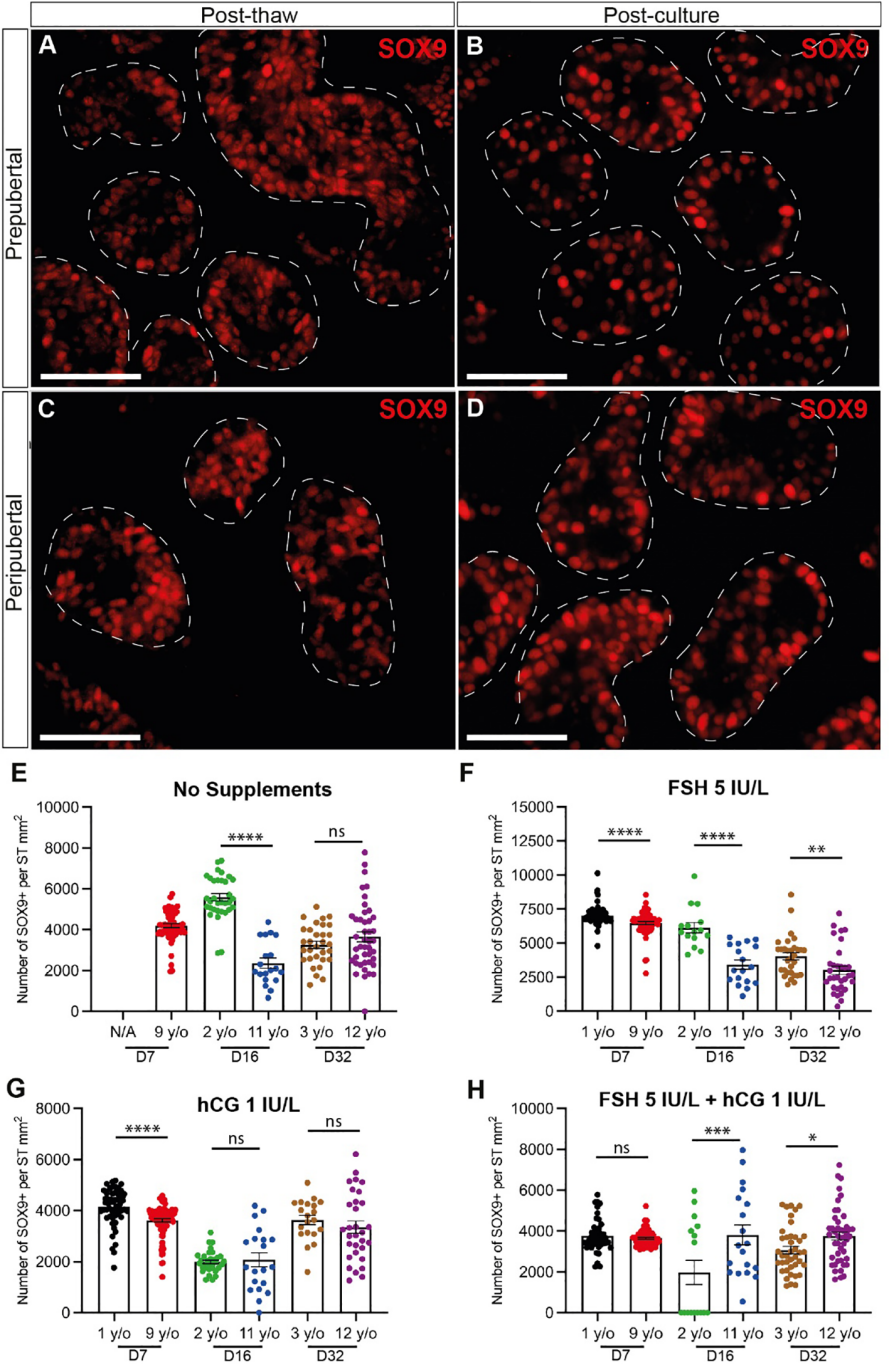
Quantification of Sertoli cells. **(A–D)** Immunofluorescence staining of SOX9+ Sertoli cells in post-thawed **(A, C)** and post-cultured **(B, D)** ITTs in prepubertal **(A, B)** and peripubertal **(C, D)** groups. Dashed white line: basal lamina. Scale bars: 50 µm. **(E–H)** Number of SOX9+ Sertoli cells per mm² of seminiferous tubule in prepubertal and peripubertal patients on day 7, 16 and 32 in four *in vitro* conditions, none **(E)**, FSH **(F)**, hCG **(G)** and FSH + hCG **(H)**. Statistically significant differences are indicated (*p ≤ 0.05, **p ≤ 0.01, ***p ≤ 0.001, ****p ≤ 0.0001). Raw data SOX9+ germ cell numbers for post-thaw day 0 as well as days 7, 16 and 32 of culture are shown in **Supplementary Table 3**.

**Figure 8:**
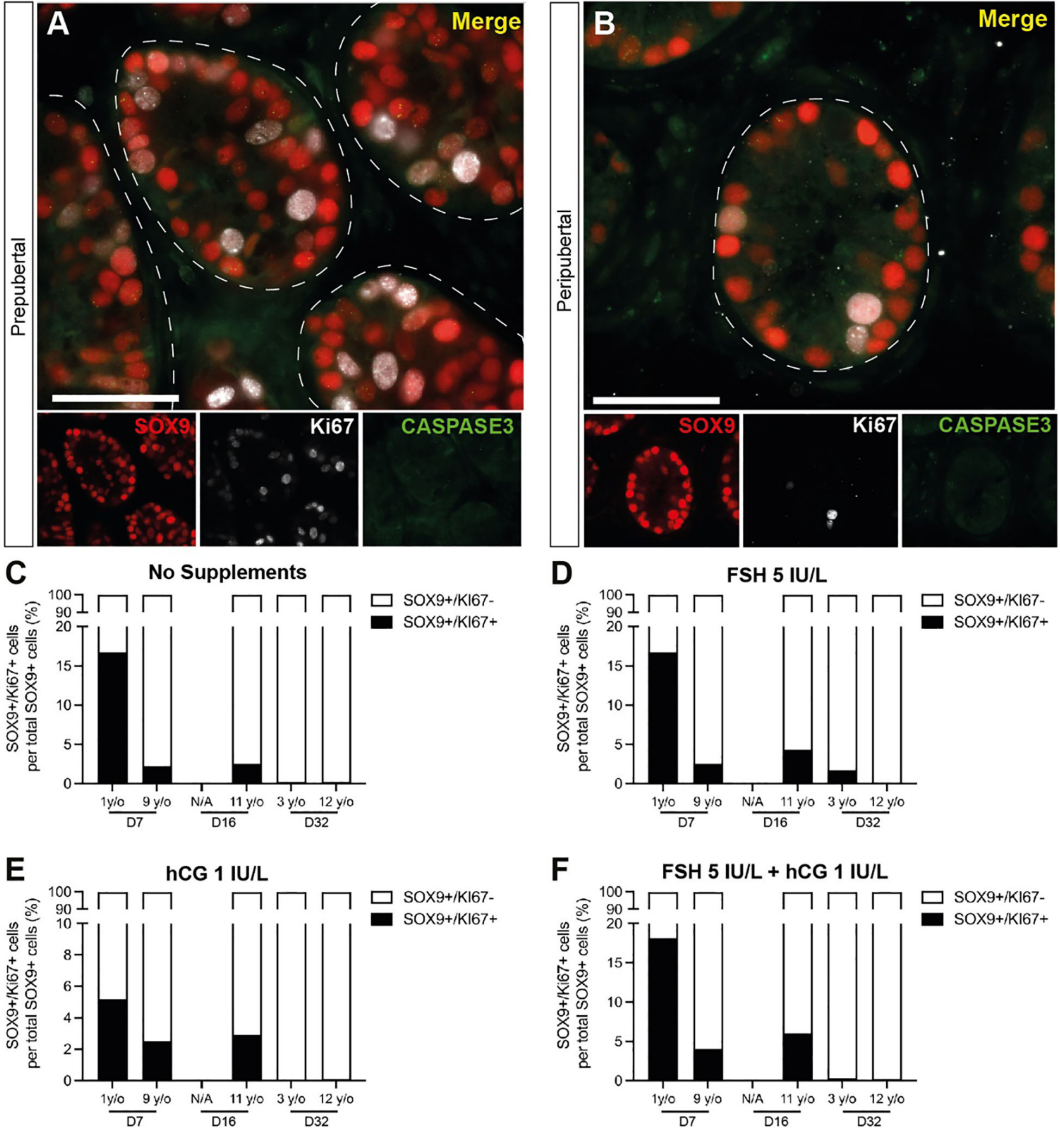
Proliferation and apoptosis of Sertoli cells. **(A, B)** Immunofluorescence co-staining of SOX9+ Sertoli cells with proliferation marker, KI67, and apoptosis marker, CASPASE3 in cultured ITTs of prepubertal **(A)** and peripubertal **(B)** groups. Dashed white line: basal lamina. Scale bars: 50 µm. **(C–F)** SOX9+/KI67+ proliferating Sertoli cells as a percentage of total SOX9+ Sertoli cells in prepubertal and peripubertal patients on day 7, 16 and 32 in four *in vitro* conditions, none **(C)**, FSH **(D)**, hCG **(E)** and FSH + hCG **(F)**. Raw quantitative data for SOX9+ Sertoli cells in each treatment condition are shown in **Figure 7**.

SOX9+ Sertoli cells were observed throughout the culture period and their numbers were not consistently impacted by age, gonadotropin treatment or time in culture (**Figure 7**). Individual values for SOX9+ cells in each treatment group at each culture timepoint, including post-thaw day 0 are in **Supplementary Table 3**. Gonadotropin treatments did appear to impact the number of SOX9+ cells, but the direction of impact was inconsistent within and between groups (**Figures 6E-H**), suggesting that changes may be due to individual variation among patient tissues. Proliferation (KI67+) and apoptosis (CASPASE3+) of Sertoli cells (SOX9+) were evaluated by immunofluorescence co-staining (**Figures 8A, B**). While the 1-year-old sample had a higher proportion of proliferating Sertoli cells in all treatment groups, there was no consistent impact of gonadotropin treatment on the proportion of proliferating Sertoli cells (**Figures 8C-F**). The proportion of proliferating Sertoli cells decreased from day 7 to day 32 in both age groups and in all conditions (**Figures 4B-H**, **Supplementary Figure 2**). The number of CASPASE3+ apoptotic Sertoli cells was low in all culture conditions and both groups (data not shown).

### Sertoli cell maturation

**Figure 9:**
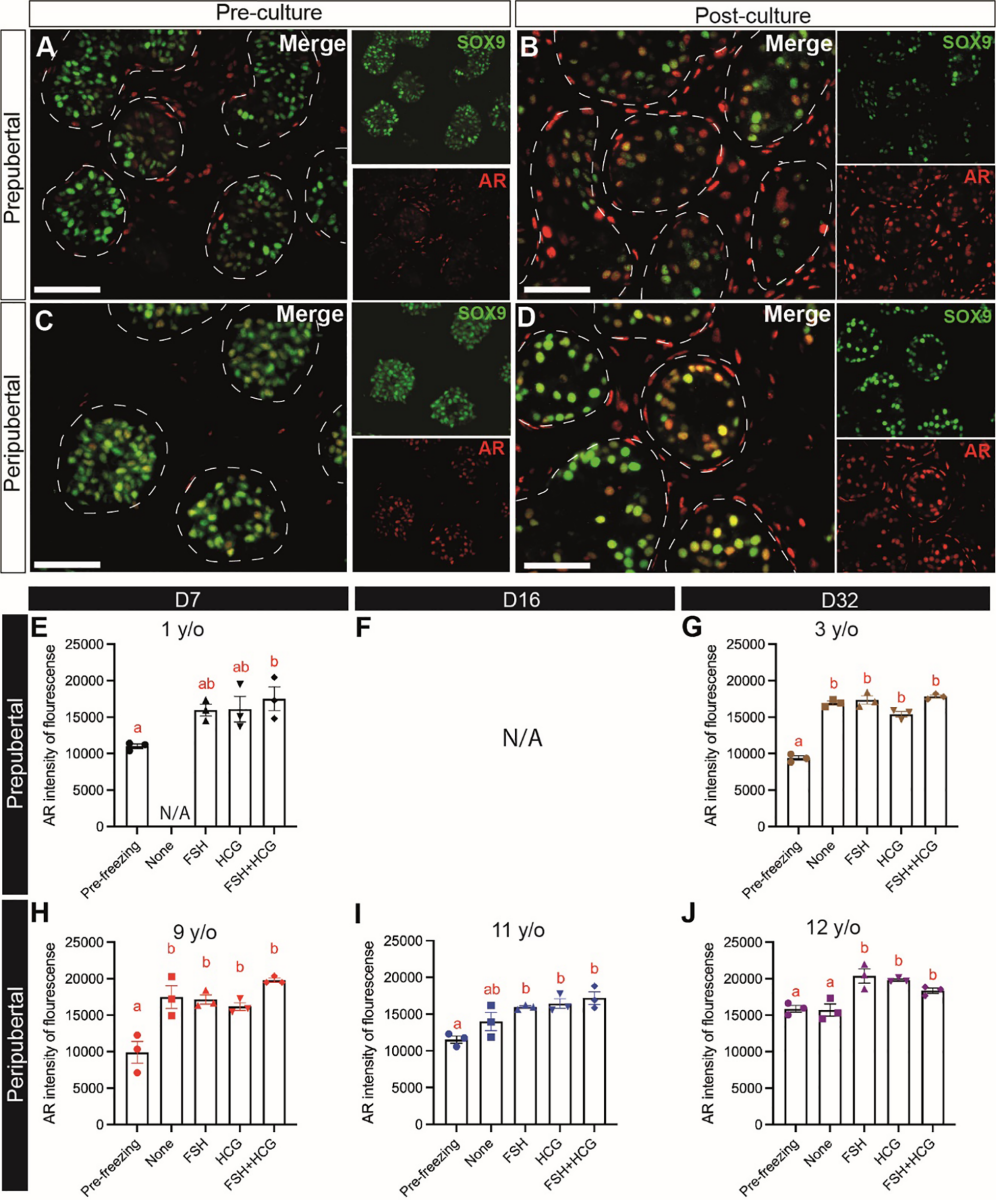
Sertoli cell maturation (AR expression). **(A–D)** Immunofluorescence co-staining of AR and SOX9+ Sertoli cells in pre-cultured **(A, C)** and post-cultured **(B, D)** ITTs in prepubertal **(A, B)** and peripubertal **(C, D)** groups. Dashed white line: basal lamina. Scale bars: 50 µm. **(E–J)** AR fluorescence intensity in prepubertal **(E–G)**, F is missing data, See **Supplementary Table 1** for explanation) and peripubertal **(H–J)** patients in four *in vitro* conditions, none, FSH, hCG and FSH + hCG at days 7 **(E, H)**, 16 **(F, I**) and 32 **(G, J)** of culture. Different red letters above the bars indicate statistically significant differences between groups (p ≤ 0.05).

Sertoli cell maturation was assessed by immunofluorescence co-staining for SOX9 and androgen receptor (AR). There were fewer AR+ Sertoli cells in the prepubertal samples (**Figure 9A**) than peripubertal samples (**Figure 9C**) prior to culture. The number (**Figures 9B, D**) and fluorescence intensity (**Figures 9E-J**) of AR+ cells increased significantly during culture in both age groups. Gonadotropin treatments did not consistently impact Sertoli cell AR expression intensity (**Figures 9E-J**).

### Tight junction protein expression

**Figure 10:**
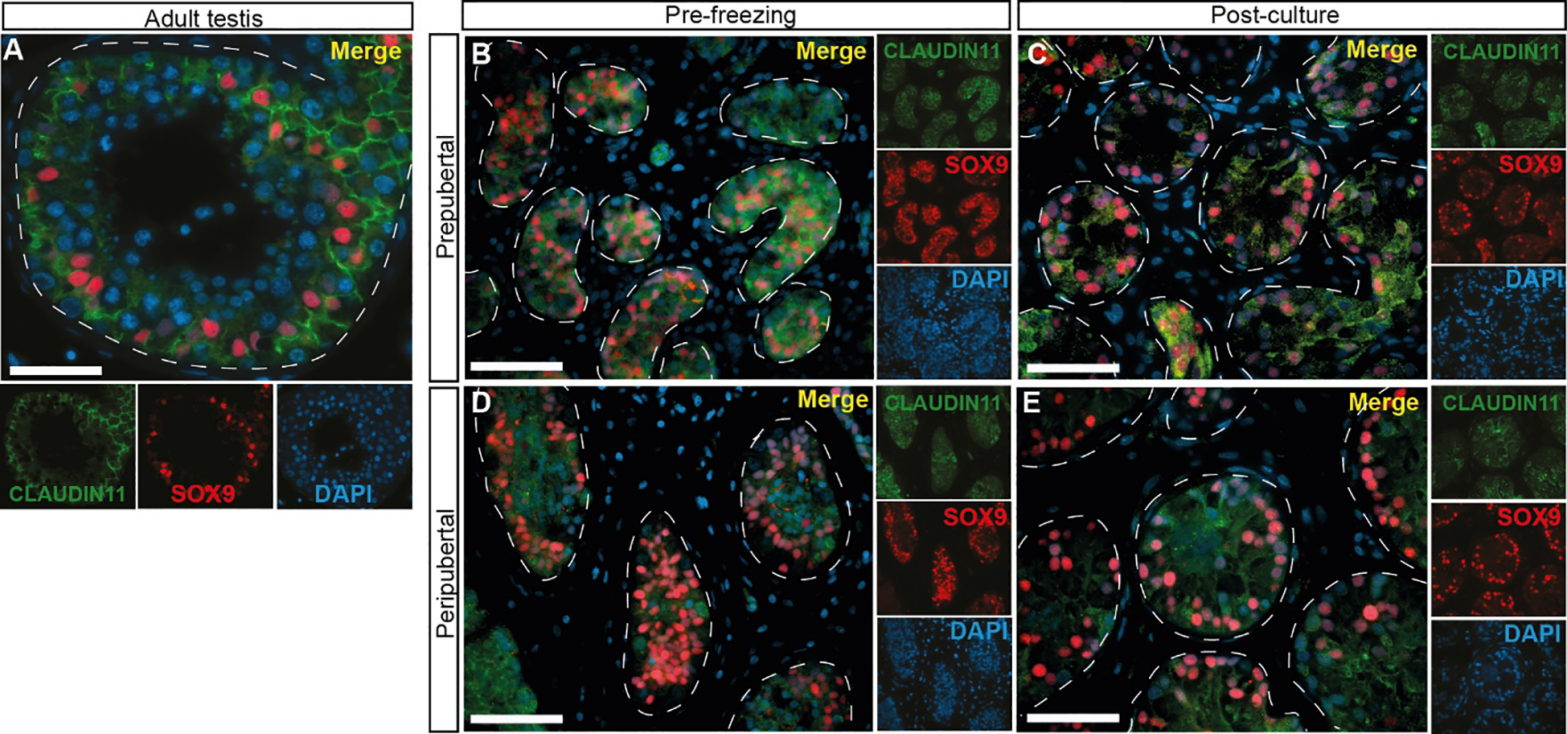
CLAUDIN 11 expression, a marker of Sertoli cell tight junctions and the blood-testis barrier. **(A–E)** Immunofluorescence staining for CLAUDIN11 and SOX9 in adult testicular tissue **(A)**, prepubertal **(B, C)** and peripubertal **(D, E)** ITTs before **(B, D)** and after **(C, E)** culture. Dashed white line: basal lamina. Scale bars: 50 µm.

CLAUDIN11 immunofluorescence was used to mark Sertoli cell tight junctions and the blood-testis barrier (BTB). CLAUDIN11 clearly delineated the BTB in adult tubules, separating the basal from the adluminal compartment of the seminiferous tubules (**Figure 10A**). In contrast, CLAUDIN11 was diffuse and did not delineate a BTB in the seminiferous tubules of ITT in either age groups, before (**Figures 10B, D**) or after (**Figures 10C, E**) culture.

### Leydig cell maturation and functionality

**Figure 11:**
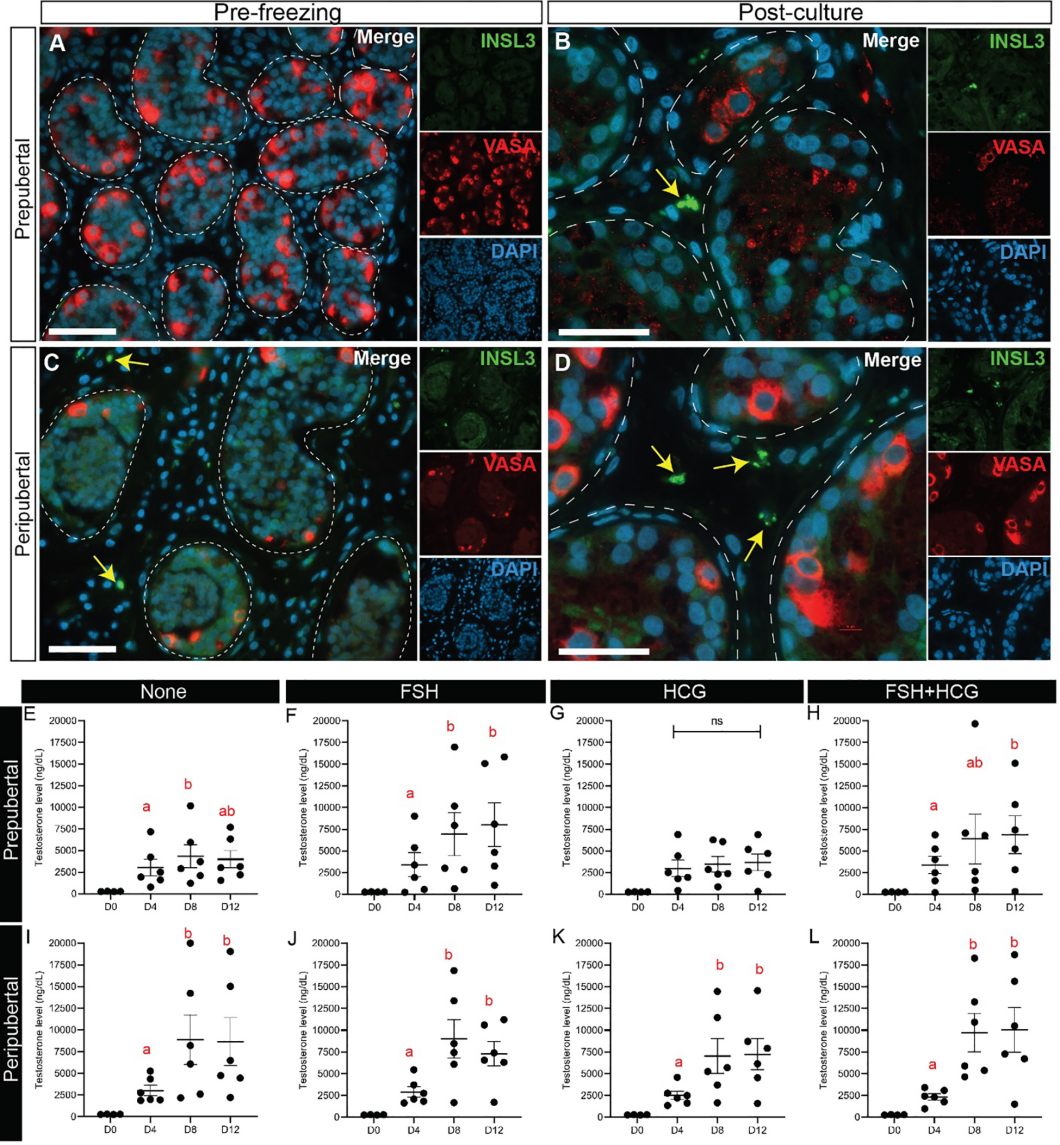
Leydig cell maturation and testosterone secretion. **(A-D)** Immunofluorescence staining for INSL3 and VASA in prepubertal **(A, B)** and peripubertal **(C, D)** patients before **(A, C)** and after **(B, D)** culture. Dashed white line: basal lamina. Scale bars: 50 µm. **(E-L)** Testosterone levels (ng/dL) in prepubertal **(E-H)** and peripubertal **(I-L)** patients in four *in vitro* conditions, none **(E, I)**, FSH **(F, J)**, hCG **(G, K)** and FSH + hCG **(H, L)** and on days 4, 8 and 12 of culture. Different red letters above the bars indicate statistically significant differences between groups (p ≤ 0.05).

INSL3+ Leydig cells were virtually absent in prepubertal, preculture tissues (**Figure 11A**), but appeared by day 32 in culture (**Figure 11B**). INSL3+ Leydig cells were present in peripubertal, preculture tissues (**Figure 11C**) as well as all culture days (**Figure 11D**). We also investigated Leydig cell functionality by measuring testosterone secretion into the media on days 4, 8 and 12 of culture. Testosterone levels in culture medium from both age groups and in most treatment, conditions were higher on days 8 and 12 of culture than in pre-culture, day 0 or culture day 4 media (**Figures 11E-L**). The FSH treatment group had significantly higher levels of testosterone on days 8 and 12 than day 4 of culture. The hCG treatment group had higher testosterone levels on days 8 and 12 than day 4 in the peripubertal groups, but not the prepubertal group. The combination FSH + hCG group had higher testosterone levels on day 12 than day 4. There was no difference in testosterone levels between the two age groups in all conditions (data not shown).

## Discussion

Immature testicular tissue has been cryopreserved for thousands of patients worldwide, dating back to 2005 (40). Our center has cryopreserved testicular tissue for >700 patients since 2011 and some of those patients are returning to use their tissue for reproduction. Autologous spermatogonial stem cell transplantation and testicular tissue grafting are mature technologies that may be ready for the clinic (15), but those technologies will not be safe or appropriate for all patients. Organotypic culture is an *ex vivo* approach to mature ITT and produce sperm. The proof in principle for this approach in mice was initially published by Sato and colleagues in 2011 with the production of sperm and offspring (21). Several labs have attempted to replicate those results with human ITT, but progress has been limited. Human ITT can remain viable for times ranging from 7-139 days in culture. Spermatogonia survive and sporadically differentiate to produce spermatocytes or spermatids, but robust regeneration of a complete seminiferous epithelium has not been described (29–32). Previous studies of human ITT organotypic culture included FSH and/or LH in some or all of their culture conditions but did not independently and exclusively test the impact of those two gonadotropins.

The pubertal surge of gonadotropins is necessary to mature testicular somatic cells and create an environment that supports the initiation of spermatogenesis (33, 34, 41, 42). Similarly, we hypothesize that LH (hCG) and/or FSH are necessary to mature somatic cells in ITT and support spermatogenesis. Surprisingly, it does not appear that these gonadotropins have been independently tested in organotypic culture of human ITT in the absence of other hormones or growth factors. We replicated the transwell ITT culture system described by de Michele and colleagues because that system was the only one to report the appearance of haploid cells (32); and tested four hormonal conditions (no hormones, 5 IU/L FSH, 1 IU/L hCG, and 5IU FSH + 1 IU/L hCG). These are the gonadotropin doses used by de Michele and colleagues. We also compared culture outcomes from prepubertal versus peripubertal testicular tissues to test the hypothesis that testicular somatic cells may already be partially matured in the peripubertal testis.

Upon thawing of cryopreserved ITT, we found that integrity of seminiferous tubules was disrupted compared to the pre-cryopreservation histology. However, tubular integrity quickly improved in culture, with tubular integrity scores resembling the pre-cryopreservation tissues at all culture times and treatment groups. Tubular integrity was maintained for up to 32 days in culture (the longest time tested in this study). As expected, VASA+ germ cells were present in higher numbers in peripubertal tissues than prepubertal tissues. VASA+ cells in both age groups were maintained throughout the culture period but their numbers declined over time in culture, as reported by others (29–32). However, gonadotropin treatments in any combination increased the number of germ cells remaining in peripubertal patient samples compared to the no treatment group, except for the 9-year-old patient, who had a higher number of germ cells in all treatment conditions. Gonadotropin treatment did not impact the number of VASA+ germ cells in prepubertal samples, perhaps suggesting that prepubertal somatic cells were not yet competent to respond to gonadotropins. Prepubertal samples tested in this study were from 1, 2 and 3 year old patients. Future studies should test older prepubertal stages (e.g., 4-8) to learn when prepubertal testicular somatic cells acquire the competence to respond to gonadotropins and dissect the molecular mechanisms that regulate that transition. Similar to VASA+ cells, UCHL1+ undifferentiated spermatogonia were present and proliferating throughout the 32 day culture period, which again, is consistent with previous reports (30, 32). Gonadotropin treatments did not appear to impact the number or proportion of proliferating spermatogonia in either age group. This is in contrast to the observations of Medrano and colleagues, who found that supplementation with gonadotropins (FSH and LH) increased the number of UTF1+ undifferentiated spermatogonia (29). Those differences may be attributed to the fact that UTF1 is a more restricted marker of undifferentiated spermatogonia, while UCHL1 has a broader expression profile that also includes some differentiating spermatogonia (43).

SYCP3 and CREM were used to assess spermatogonial differentiation to spermatocytes and spermatids, respectively. Neither marker was present in pre-culture tissues or day 7 cultures from either age group. SYCP3+ spermatocytes began to appear in both age groups on days 16 and 32 of culture, which is consistent with previous reports, *in vivo* and *in vitro* (32, 44, 45). There was no consistent effect of gonadotropin treatments on the appearance of SYCP3+ cells. We did not observe CREM+ spermatids in either age group, at any time of culture in any of the culture conditions. Perhaps that is not surprising, since the process of spermatogenesis from spermatogonia to sperm takes 74 days in humans (46) and we did not extend our cultures to 74 days. Extended culture periods may allow sufficient time for progression through meiosis and production of haploid cells. We note, however, that de Michele and colleagues (32), using the same culture system, observed haploid cells by chromatin *in situ* hybridization on days 16, 32, 64 and 139 of ITT organotypic culture. The authors noted, however, that ACE+ spermatids were observed by immunohistochemistry only in one out of 40 sections in a 64-day culture from one patient and expression of late spermatid markers TP1 and PRM2 were not observed (32). While some studies indicated that spermatogenesis occurs on schedule in rodent organotypic culture (21, 47, 48), others have suggested that *in vitro* spermatogenesis may occur at an accelerated pace in mouse and humans (48, 49).

Sertoli cells are the testicular somatic cells of the seminiferous tubules that switch from immature and proliferative cells to mature and non-proliferative status around the time of puberty under the control of FSH and testosterone (34). They are considered the nurse cells of the adjacent germ cells since they orchestrate every stage of germ cell development from the most undifferentiated spermatogonia to the most differentiated spermatozoa. Sertoli cells also mediate many of the effects of testosterone on germ cell development (36). We found that SOX9+ Sertoli cell numbers, normalized to area of seminiferous tubule, were present in similar numbers in prepubertal and peripubertal samples and maintained throughout the 32 day culture period. Sertoli cell numbers were not impacted by gonadotropin treatment. This is consistent with the results of de Michele and colleagues who found that Sertoli cell numbers were maintained in long-term culture in the presence of FSH or FSH + hCG and other factors (32); but differs from the results of Medrano and colleagues, who found that Sertoli cell numbers decreased by 70 days in culture in the presence of FSH and hCG. While Sertoli cell number remained relatively constant throughout the culture period in our study, Sertoli cell proliferation decreased over time in both age groups, which may be an indicator of Sertoli cell differentiation. Sertoli cell differentiation was also indicated by increased AR expression during culture, consistent with previous reports (31, 32), and this occurred in a gonadotropin independent manner. Portela and colleagues did not observe induction of AR expression in ITT organotypic culture. Moreover, maturation of Sertoli cells in our study was likely incomplete because CLAUDIN11 proteins did not organize to delineate a BTB as shown in the adult control (**Figure 10A**). A functional BTB is necessary for the maintenance of spermatogenesis, *in vivo* (50). Others have also reported expression of BTB proteins (ZO-1, CLAUDIN11, CONNIXIN43) in ITT organotypic culture. Medrano and colleagues reported that ZO-1 expression pattern was chaotic (29). de Michele and colleagues reported that CLAUDIN 11 expression was constant for all patients at all culture times while CONNEXIN 43 expression was observed from day 16 onwards (51). *in vitro*

INSL3 is a constitutive marker of Leydig cell differentiation (52). As expected, we found that INSL3 expression was absent in prepubertal testis tissues, but present in peripubertal testis tissues prior to culture. INSL3 expression was observed in the testicular interstitium of both prepubertal and peripubertal tissues by 32 days in culture and this was not gonadotropin dependent (not shown). The number of INSL3+ cells was not quantified in this study but appeared fewer in number in the cultured samples than in the positive control (**Supplementary Figure 3**), perhaps indicating that Leydig cell differentiation was incomplete. Nonetheless, all tissues in all treatment conditions secreted testosterone into the culture medium, which increased over time in culture in most samples. The exceptions were untreated and hCG treated prepubertal tissues that did not exhibit increased testosterone secretion over time. This may indicate that FSH was needed to mature the Leydig cells and enhance responsiveness to hCG. This interpretation is consistent with a previous report of Kerr and Sharpe (35), who reported that FSH and not LH was required to mature Leydig cells and enhance hCG stimulated testosterone production in whole testes or dispersed Leydig cells.

## Conclusion

We found that cryopreserved ITT from patients could be maintained in organotypic culture for up to 32 days. Testicular germ cells and somatic cells remained viable throughout the culture period. We observed sporadic differentiation of spermatogonia to produce SYCP3+ spermatocytes but did not observe differentiation to CREM+ meiotic or post-meiotic cells and did not observe the establishment of a complete seminiferous epithelium. Our study revealed differences in prepubertal and peripubertal tissues in terms of somatic differentiation and responsiveness to gonadotropins (testosterone production), but these differences did not impact the extent of spermatogenesis achieved in culture. Higher doses of gonadotropins and/or longer culture periods may support more complete spermatogenic lineage development in future studies. Somatic differentiation, especially of prepubertal tissues, occurred during culture (increased AR and INSL3 expression), but differentiation of Sertoli cells and Leydig cells may have been incomplete, which may have impacted the extent of spermatogenesis achieved in this study. The main limitations in this study were the low number of patients and the tiny size of each ITT fragment available to research. This made it impossible to spread the tissue from a single patient across all treatment groups and all culture timepoints. Each timepoint within each treatment group is represented by only one patient. Therefore, some of the observed differences were due to variations among individual patients, which may have obscured treatment effects. Nonetheless, our results were consistent with previous reports indicating that ITT can be maintained in culture while highlighting limitations that will be the focus of ongoing studies to achieve complete and robust spermatogenesis in cultured human testicular tissues.

## Data availability statement

The original contributions presented in the study are included in the article/**Supplementary Material**. Further inquiries can be directed to the corresponding author.

## Ethics statement

The studies involving humans were approved by University of Pittsburgh Institutional Review Board. The studies were conducted in accordance with the local legislation and institutional requirements. Written informed consent for participation in this study was provided by the participants’ legal guardians/next of kin. The animal study was approved by University of Pittsburgh Institutional Animal Care and Use Committee. The study was conducted in accordance with the local legislation and institutional requirements.

## Author contributions

NY, SA and KO designed the experiments. NY conducted the experiments and wrote the manuscript. NY, AC-B, SA and KO analyzed and interpreted the results. TC conducted statistical analysis. NY, AC-B, SA and KO revised the manuscript. All authors contributed to the article and approved the submitted version.
