# Supplementary material for: Human immature testicular tissue organ culture: a step towards fertility preservation and restoration
Source: Front Endocrinol (Lausanne). 2023 Aug 28;14:1242263. doi: 10.3389/fendo.2023.1242263 (PMC10494240; doi:10.3389/fendo.2023.1242263)

Supplementary Figure 1. Quantification of VASA+ germ cells during culture. (A-H). Number of VASA+ germ cells per mm² of seminiferous tubule in prepubertal (A-D) and peripubertal (E-H) patients on days 7, 16 and 32 of culture in four *in vitro* treatment conditions, none (A, E), FSH (B, F), hCG (C, G) and FSH + hCG (D, H). Different red letters above the bars indicate statistically significant differences between groups ($p \leq 0.05$).

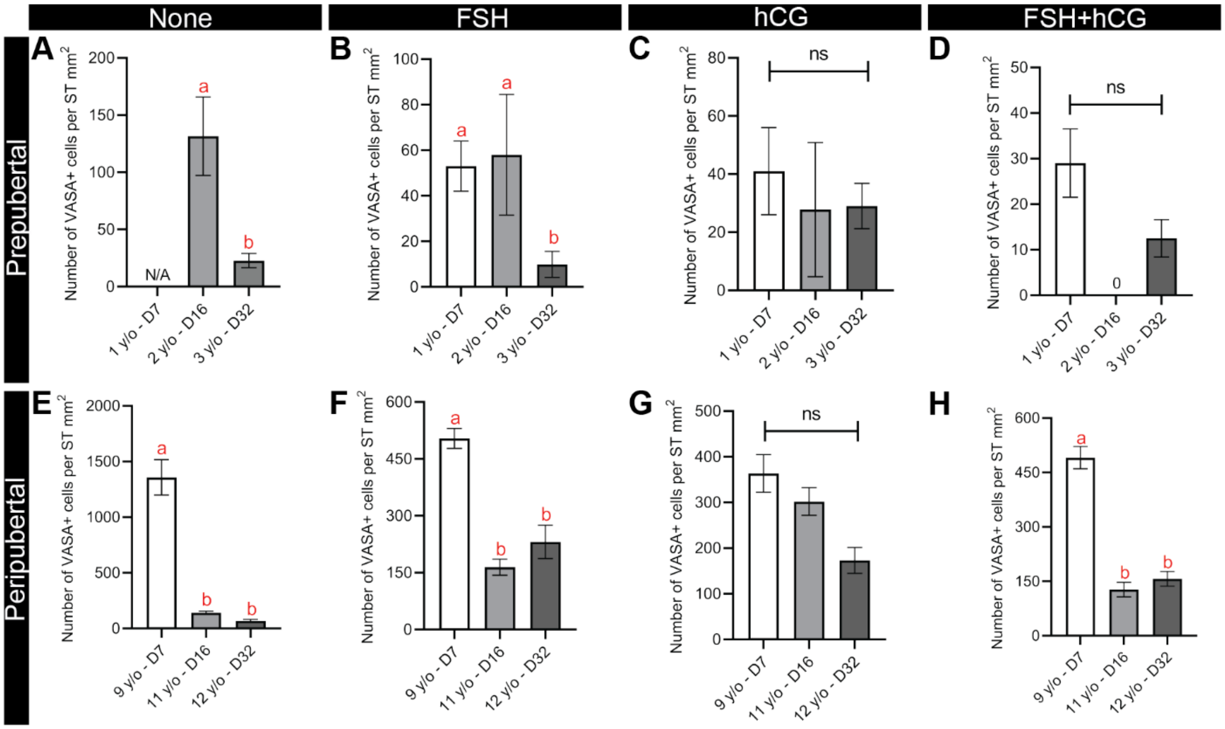

Supplementary Figure 2. Quantification of proliferating Sertoli cells over time in culture. (A-H). Number of SOX9+/KI67+ proliferating Sertoli cells per mm² of seminiferous tubule in prepubertal (A-D) and peripubertal (E-H) patients on day 7, 16 and 32 of culture in four *in vitro* treatment conditions, none (A, E), FSH (B, F), hCG (C, G) and FSH + hCG (D, H). Different red letters above the bars indicate statistically significant differences between groups ($p \leq 0.05$).

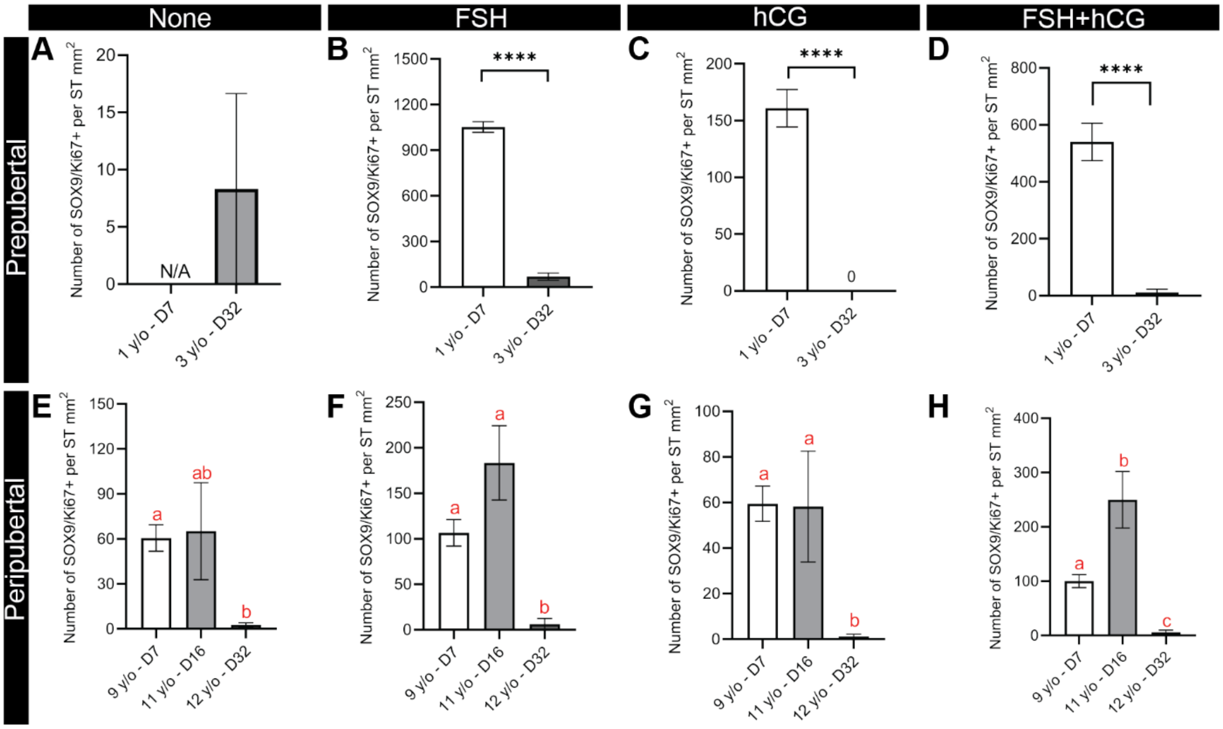

Supplementary Figure 3. (A) Negative controls for each marker used in the study. **(B)** Positive control staining of adult human testicular tissues (containing complete spermatogenesis) for each marker used in this study. Scale bar: 50 µm.

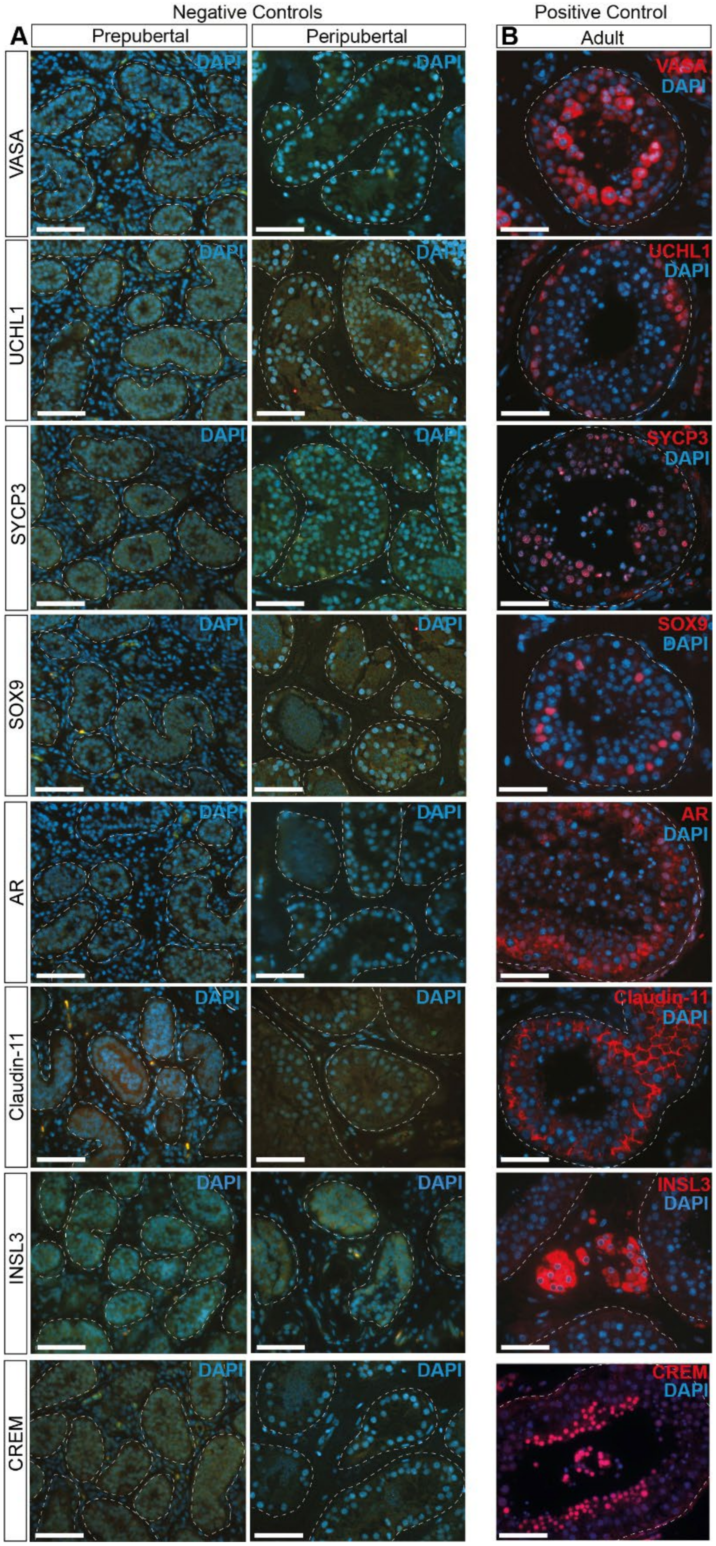

Supplement: Supplementary file 1 [file Image_1.pdf]
