# Supplementary material for: Human immature testicular tissue organ culture: a step towards fertility preservation and restoration
Source: Front Endocrinol (Lausanne). 2023 Aug 28;14:1242263. doi: 10.3389/fendo.2023.1242263 (PMC10494240; doi:10.3389/fendo.2023.1242263)
Supplement: Supplementary file 2 [file Table_1.docx]

| **Supplementary Table 1. Explanation of missing data (N/A)** | | | | |
| --- | --- | --- | --- | --- |
| **Figure** | **Patient** | **condition** | **Time point** | **Reason** |
| All figures | P1 (1y/o) | None | D7 | Lost in histology preparation |
| 2, 3, 5, 6 | P2 (2 y/o) | Post thaw | Before culture | Original fragment size was small and not sufficient for all treatment groups |
| 4, 7, 8, 9 | P2 (2 y/o) | All conditions | Day 16 | No more tissue left for sectioning and analysis |

**Supplementary Table 2: Patient groups for testosterone assay**

| **Pubertal category** | **Patient** | **Age (years)** | **Diagnosis** | **Previous treatment** |
| --- | --- | --- | --- | --- |
| Prepubertal | 1* | 2 | Chronic granulomatous disease | None |
|  | 2 | 1 | Wiskott-Aldrich syndrome | None |
|  | 3 | 5 | Sickle cell anemia | None |
|  | 4* | 3 | X linked chronic granulomatous disease | None |
|  | 5 | 1 | Sickle cell anemia | None |
|  | 6 | 2 | Anaplastic Wilms tumor | None |
| Peripubertal | 7* | 11 | Chronic granulomatous disease | None |
|  | 8 | 9 | Chronic granulomatous disease | None |
|  | 9 | 9 | Sickle cell anemia | None |
|  | 10* | 12 | Ewing’s sarcoma of fibula | None |
|  | 11 | 11 | Metastatic neuroblastoma | None |
|  | 12 | 12 | Ewing's sarcoma of fibula | None |

*Patients 1, 4, 7 and 10 correspond to P2, P3, P5 and P6 respectively from table 1.

**Supplementary Table 3**
